# Down-Regulation of mir-221 and mir-222 Restrain Prostate Cancer Cell Proliferation and Migration That Is Partly Mediated by Activation of SIRT1

**DOI:** 10.1371/journal.pone.0098833

**Published:** 2014-06-03

**Authors:** Xiao Yang, Yingmei Yang, Rong Gan, Lingxu Zhao, Wei Li, Huaibin Zhou, Xiaojuan Wang, Jianxin Lu, Qing H. Meng

**Affiliations:** 1 Wenzhou Medical University School of Laboratory Medicine and Life Sciences, Wenzhou, Zhejiang, China; 2 Key Laboratory of Laboratory Medicine, Ministry of Education, Wenzhou, Zhejiang, China; 3 Zhejiang Provincial Key Laboratory of Medical Genetics, Wenzhou, Zhejiang, China; 4 Department of Clinical Laboratory Medicine, Jinhua People's Hospital, Jinhua, Zhejiang, China; 5 Department of Laboratory Medicine, The University of Texas MD Anderson Cancer Center, Houston, Texas, United States of America; University of Texas, MD Anderson Cancer Center, United States of America

## Abstract

Studies have shown that miR-221 and miR-222 are deregulated in many cancers, including prostate cancer. Nevertheless, the biological role and the underlying mechanisms of miR-221 and miR-222 in the pathogenesis of androgen-independent prostate cancer are still not clear. The proliferation, apoptosis, cell cycle distinction, and migration capacity of prostate cells were determined following transfection of miR-221 or miR-222 inhibitor. The biological impact and regulation of SIRT1 on prostate cancer cells were investigated. MiR-221 and miR-222 were highly expressed in PC-3 cells compared with in LNCap cells. After miR-221 or miR-222 expression was inhibited, the proliferation and migration rates of PC-3 cells decreased and the apoptosis rate increased. Moreover, SIRT1 protein was up-regulated in cells after they were transfected with miR-221 or miR-222 inhibitor. Cells transfected with siSIRT1 showed increased migration and a decreased apoptosis rate, but there was no significant effect on cell proliferation compared with the controls. There was a negative correlation between miR-221 or miR-222 and SIRT1, but no direct target relationship was identified. These data demonstrate that miR-221 and miR-222 are highly expressed in PC-3 cells. Their inhibition leads to reduced cell proliferation and migration and increased apoptosis in prostate cancer cells. These effects are potentially mediated by up-regulation of SIRT1.

## Introduction

Prostate cancer (PCa) is one of the most common malignancies and the second leading cause of cancer death for male in the western world. Approximately 238,590 new cases were diagnosed in 2013 [1]. Most PCas grow slowly and are dependent on androgen for growth; thus, they respond to androgen deprivation treatment (ADT). ADT is effective, but most patients' disease will eventually become refractory and progress from androgen-dependent PCa to androgen-independent (castration-resistant) PCa, which brought great challenges to the treatment of PCa [2]. Thus, identifying a new and effective therapeutic approach has become the focus in the fight against PCa.

MicroRNAs (miRNAs) are small (approximately 21–23 nucleotides), non-protein-coding RNAs that function as post-transcriptional regulators of target genes. These molecules are mainly found in eukaryotes and are fully or partially integrated, in a complementary manner, with the target mRNA 3′UTR, resulting in the degradation or translation inhibition of target mRNA. miRNA functions in the transcriptional and post-transcriptional regulation of gene expression, influencing many cellular biological processes [3]. miRNAs are involved in multiple cell differentiation, proliferation, and apoptosis processes that are closely related to tumorigenesis [3]. Recently, some aberrantly expressed miRNAs were discovered in PCa and other cancers, indicating that they play a critical role in the molecular mechanism of cancer pathogenesis and progression [2],[4]–[8]. Furthermore, studies have shown that miR-221 and miR-222 are deregulated in many cancers, including PCa [9]–[14], and the two miRNAs play an important role in tumorigenesis and progression from androgen-dependent PCa to androgen-independent PCa [15]–[17]. Nevertheless, the results are inconsistent and even controversial and the underlying mechanisms are still not clear.

In mammals, silent information regulator 1 (SIRT1) is a member of the sirtuin family and has been shown to be highly homologous with SIRT2 in yeast [18]. SIRT1 is also known as NAD-dependent histone deacetylase and is involved in the regulation of many physiological processes, such as cell proliferation, the inflammatory response, the cell cycle, and cell migration [19]. However, it is unknown whether SIRT1 acts as a promoter gene or suppressor gene because of its complexity [20]–[23]. Its role in cancer has not been well defined. For instance, SIRT1 showed anti-oncogene action and its expression level was associated with prognosis in colon cancer [24],[25]. Nevertheless, it is considered an oncogene in breast cancer [26]. In PCa, however, the role of SIRT1 is still controversial [27],[28].

In this study, we investigated their regulatory role of miR-221 and miR-222 and their potential molecular mechanisms in PCa by transfecting miR-221 or miR-222 inhibitor in PCa cells.

## Materials and Methods

### Cell culture and plasmid transfection

Human PCa PC-3 cells (androgen-independent) and LNCap cells (androgen-dependent) were purchased from the Institute of Biochemistry and Cell Biology, Chinese Academy of Sciences. Cells were maintained in F12 (Gibco, Carlsbad, CA) containing 10% fetal bovine serum (FBS) (Gibco, Carlsbad, CA) at 37°C in a 5% CO2 atmosphere.

The pcDNA3.1-empty vector (pEX-5), pcDNA3.1-hsa-miR-221 inhibitor sponges (miR-221 inhibitor), pcDNA3.1-hsa-miR-222 inhibitor sponges (miR-222 inhibitor), pGPU6-empty (pGPU6), and pGPU6-siSIRT1 (siSIRT1) were synthesized by GenePharma (Shanghai, China). The wild-type SIRT1 3'UTR region was constructed into psiCHECK-2 by GenePharma (Shanghai, China). PC-3 cells were grown to 80%–90% confluence and transfected with plasmids using Lipofectamine 2000 (DNA/Lipofectamine 2000 = 1/2) according to the manufacturer's instructions. Four hours after transfection, the culture medium was replaced with fresh F12 containing 10% FBS. A stable transfection expression of cell lines was established after cells had been incubated in complete F12 medium with G418 (1000 µg/ml) for 15 days. We verified the clones using western blot and real time PCR, and pooled the successful clones for the experiments. The verification results of western blot are shown in Figure S1. In addition, all the experiments were also confirmed by transient transfection.

### Cell proliferation and cell cycle assay

For the cell proliferation assay, we seeded PC-3 cells with established stable expression (pEX-5, miR-221 inhibitor, miR-222 inhibitor, pGPU6, or siSIRT1) in 96-well microplates at a density of 2×10^3^ cells per well and incubated them for various periods of time (1 to 7d). After that, 10 µl of Cell Counter Kit-8 (CCK-8) reagent to each well and incubated cells at 37°C for 1.5 h. Absorbance was measured at 450 nm using an electroluminescence immunosorbent assay (ELISA) reader.

For the cell cycle assay, transfected cells were seeded in 12-well plate for 48 h. Cells in each well were collected and fixed with 70% ice-cold ethanol for 24 h at −20°C. After being washed with phosphate-buffered saline (PBS) three times, they were incubated with RNase for 1 h on ice and stained with propidium iodide for 15 min on ice. The DNA distribution was measured by flow cytometry in 4 h.

### Apoptosis assay

Stably transfected cells were seeded in 12-well plates and cultured for 48 h. Cells were then harvested and collected by centrifugation at 2000 rpm. After being washed with PBS three times, they were stained with Annexin V-enhanced green fluorescent protein (FITC) and propidium iodide (PI) for 15 min, protected from light. The cell apoptosis rate was determined by flow cytometry in 1 h.

### Wound healing assay and transwell migration assay

A wound healing assay was performed to mimic cell migration [26]. In brief, transfected cells were plated in a six-well plate at a density of 1×10^6^ cells per well. Cells were grown to 80% confluence and several wound lines were scratched vertically to the bottom with a 200- µl pipette tip. After being washed with PBS three times, cells were incubated in growth medium containing 1.5% serum. The wound width was determined every 24 h over a period of 72 h at ×100 under a Nikon Eclipse TS100 microscope (Nikon, Japan). Values were expressed as the percentage of wound closure, which was calculated as follows: percentage of wound closure  = 1− (width*_t_*/width*_0_*) ×100%.

For the cell transwell migration assay, cells were starved for 24 h, resuspended with serum-free F12 medium, and seeded (5×10^4^ cells) into a 24-well transwell with an 8- µm pore membrane insert (Corning, USA). F12 medium, supplemented with 10% FBS, was placed in the lower chamber as a chemoattractant. Cells were incubated for 72 h. Cells that penetrated the membrane were fixed with 4% paraformaldehyde for 15 min, stained with crystal violet for 30 min at ambient temperature, and photographed under a microscope (Nikon, Japan). Invaded cells were eluted down with acetic acid. Absorbance was measured at 590 nm in an ELISA reader.

### Reverse transcription and real-time polymerase chain reaction

Forty-eight hours after transfection, total RNA was extracted from cultured cells using TRIzol (Invitrogen, USA) following the manufacturer's protocols. For miRNA and SIRT1 reverse transcription, 500 ng of total RNA were reverse transcribed to cDNA with miRNA-specific RT primers (RiboBio, China) and random primer (TaKaRa, Japan), respectively. Gene expression was measured by real-time quantitative polymerase chain reaction (PCR) using an Applied Biosystems 7500 Fast Sequence Detection System and SYBR Green PCR Kit (QIAGEN, Germany) under the following conditions: denaturation at 95°C for 5 minutes, followed by 40 cycles of denaturation at 95°C for 10 sec and annealing and extension at 60°C for 30 sec. The relative miRNA and mRNA expression levels were normalized by U6 and β-actin, respectively.

### Western blot analysis

Seventy-two hours after transfection, cells were harvested and lysed in the presence of a protease inhibitor cocktail and centrifuged at 12,500 rpm for 20 minutes at 4°C. The supernatant fraction was collected and the protein concentration was measured using a bicinchoninic acid protein assay kit (Shenggong Bio-Tech Co, Ltd, Shanghai, China). An aliquot of 80 µg of denatured protein from each sample was applied to 10% sodium dodecyl sulfate polyacrylamide gel electrophoresis and transferred onto nitrocellulose membranes. Membranes were blocked with 5% skim milk for 2 h at ambient temperature, followed by incubation with primary antibody (1∶2000 dilution, Abcam, USA) at 4°C overnight and horseradish peroxidase-conjugated secondary antibody (1∶1000 dilution, Abcam, USA) for 1 h at ambient temperature. The blots were then incubated with enhanced chemiluminescence for 2 min. The signals were detected and quantified by densitometry using Quantity One software. β-actin was used as an endogenous control to normalized expression data.

### SIRT1 target prediction and luciferase activity assay

miRNA targets were predicted using the miRanda database (http://www.microrna.org/). For the luciferase activity assay, nucleotides 2341 to 2731 (the complete predicted miR-221 and miR-222 target site of the SIRT1-3′UTR) were inserted downstream of the Renilla luciferase gene in a Renilla/firefly luciferase reporter plasmid, psiCHECK-2 (GenePharma, Shanghai, China). PC-3 cells were transfected with 0.5 µg of reporter plasmids per well plus miR-221 or miR-222 inhibitor plasmid. Forty-eight hours after transfection, Renilla/firefly luciferase activity was measured by dual luciferase reporter assay (Promega, USA) in an automatic microplate reader (Thermo, USA).

### Statistical analysis

All statistical analyses were performed using SPSS software version 17.0 (Chicago, Illinois, USA). Data were expressed as the mean value ± SEM. Statistical significance was determined by an analysis of variance or two-tailed Student's t-test. A p value <0.05 was considered statistically significant. All experiments were performed at least three times.

## Results

### Expression of mir-221 and mir-222 in pca cells

**Figure 1:**
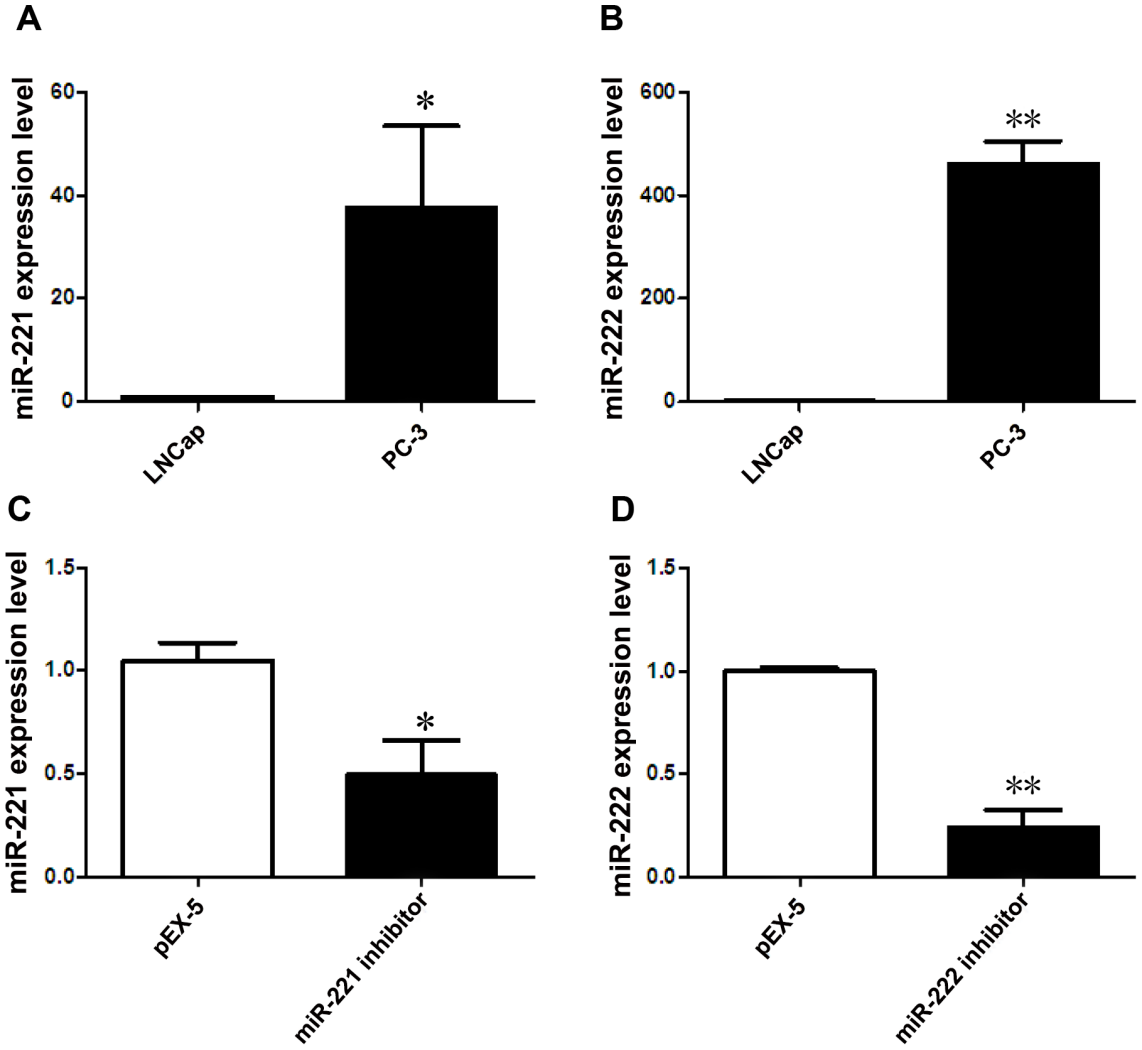
miR-221 and miR-222 expression in PCa cells. (**A**) miR-221 levels in PC-3 and LNCap cells. (**B**) miR-222 levels in PC-3 and LNCap cells. (**C**) miR-221 levels in PC-3 cells after transfection with pEX-5 (empty vector plasmid) or miR-221 inhibitor. (**D**) miR-222 levels in PC-3 cells after transfection with pEX-5 or miR-222 inhibitor. Data represent the mean value ± SEM from three separate experiments. ^*^P<0.05,^**^P<0.01.

miR-221 was highly expressed in PC-3 cells compared with LNCap (P<0.05) (Figure 1A). Similarly, miR-222 was also markedly increased in PC-3 cells compared with LNCap (P<0.01) (Figure 1B). Transfection with miR-221 inhibitor dramatically suppressed the expression of miR-221 in PC-3 cells (P<0.05) (Figure 1C). Likewise, miR-222 expression was significantly decreased after transfection of the miR-222 inhibitor (P<0.01) (Figure 1D).

### Effects of mir-221 inhibitor and mir-222 inhibitor on cell proliferation, cell cycle distribution, and apoptosis in pc-3 cells

**Figure 2:**
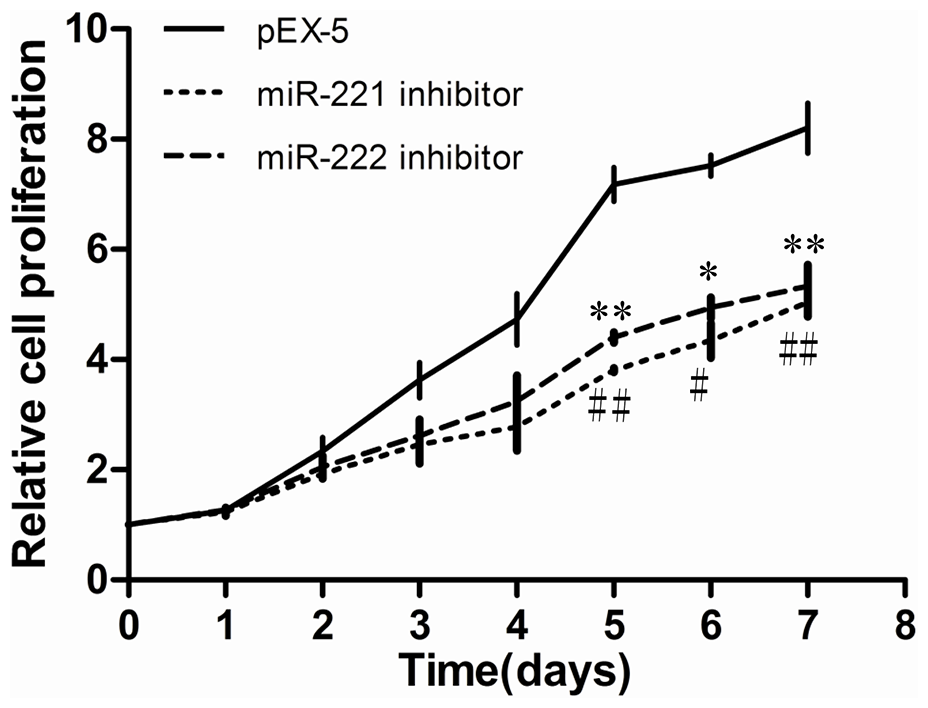
Effects of miR-221 inhibitor and miR-222 inhibitor on PC-3 cell proliferation. PC-3 cells were transfected with empty vector plasmid (pEX-5), miR-221 inhibitor, or miR-222 inhibitor. Cell proliferation was analyzed using a CCK-8 assay. The proliferation of cells transfected with miR-221 inhibitor or miR-222 inhibitor was reduced compared with that transfected with pEX-5. Data represent the mean value ± SEM from three separate experiments. ^*,#^ P<0.05 vs pEX-5, ^**,##^ P<0.01 vs pEX-5.

As shown in Figure 2, compared with cells transfected with empty vector (pEX-5), cells transfected with miR-221 inhibitor or miR-222 inhibitor showed significantly reduced proliferation on the fifth, sixth, and seventh days (P<0.01 to 0.05).

**Figure 3:**
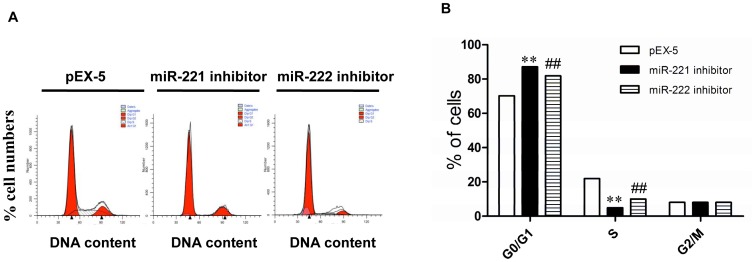
Effects of miR-221 inhibitor and miR-222 inhibitor on PC-3 cell cycle distribution. (**A**) Cell DNA content distribution in each phase. (**B**) Percentage of cells distributed in each phase of the cell cycle. **,^##^ P<0.01 vs pEX-5.

A flow cytometry analysis revealed that 70.2±1.8% of cells transfected with pEX-5 were in the G0/G1 phases, whereas 87.1±2.0% and 81.9±0.9% of cells transfected with miR-221 inhibitor and miR-222 inhibitor were in G0/G1, respectively (P<0.01) (Figures 3 A and B). Transfection with miR-221 inhibitor or miR-222 inhibitor resulted in a much lower percentage of cells in S phase compared with those transfected with pEX-5 (4.9±1.9% or 9.9±1.1% vs. 21.9±1.7%, P<0.01). There were no significant differences in cells in G2/M phases among the groups.

**Figure 4:**
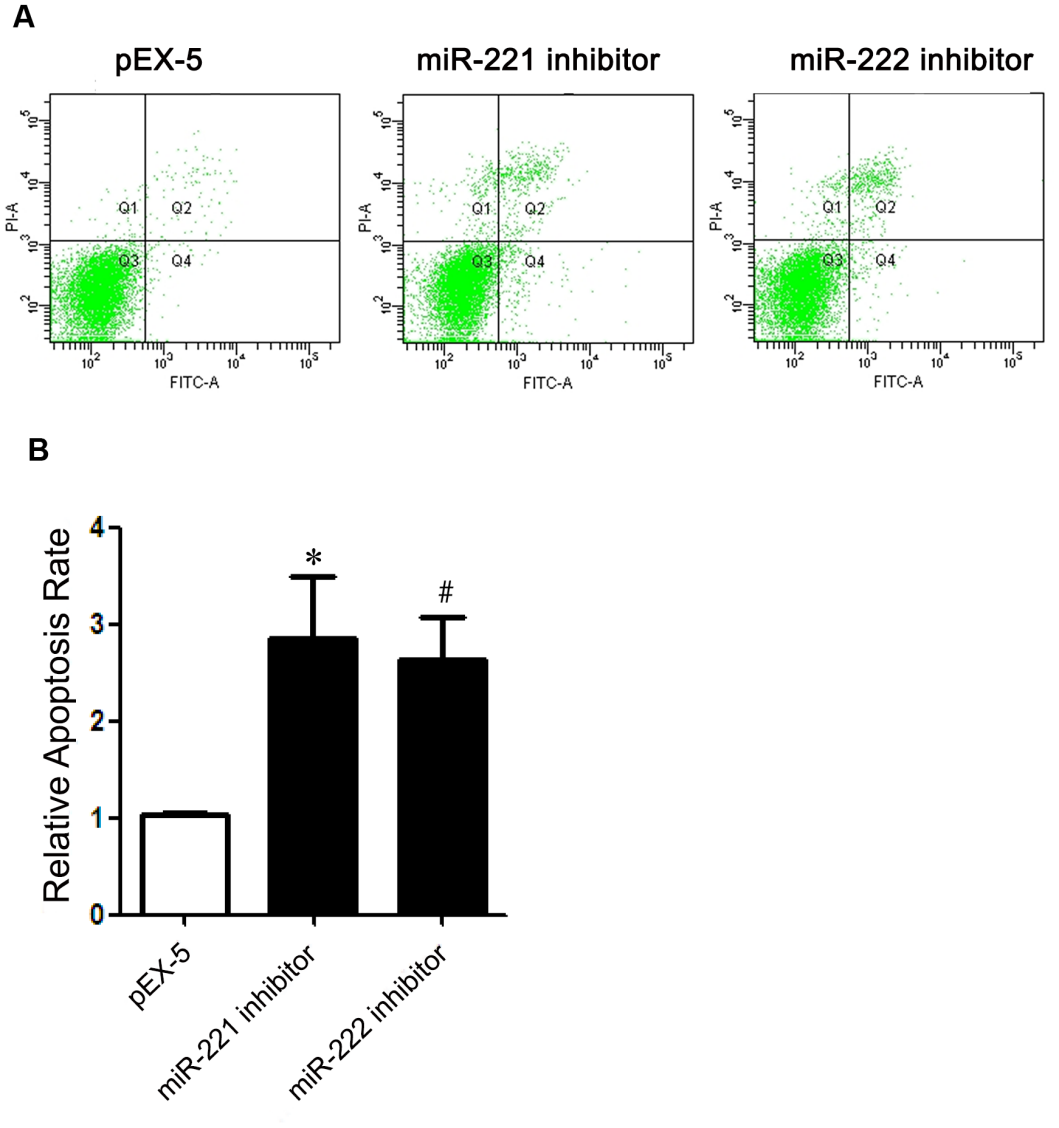
Effects of miR-221 inhibitor and miR-222 inhibitor on apoptosis. (**A**) Apoptotic cell distribution of PC-3 cells transfected with pEX-5 miR-221 inhibitor or miR-222 inhibitor by flow cytometry. (**B**) Relative apoptosis rate of each group. Data represent the mean value ± SEM from three separate experiments.(^*,#^ P<0.05 vs pEX-5.

The apoptosis rates of PC-3 cells transfected with miR-221 inhibitor or miR-222 inhibitor were increased by 2.8-fold and 2.6-fold, respectively, compared with those transfected with pEX-5 (P<0.05) (Figures 4A and B).

### Effects of mir-221 inhibitor and mir-222 inhibitor on pc-3 cell migration

**Figure 5:**
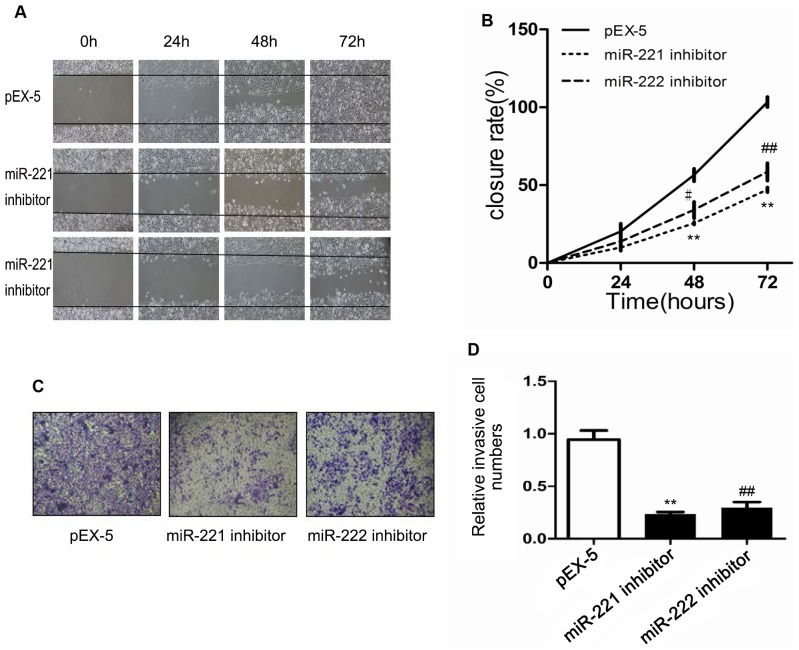
Effects of miR-221 inhibitor and miR-222 inhibitor on PC-3 cell migration. (**A**) Wound healing assay shows the closure rates of cells transfected with pEX-5 (empty vector plasmid), miR-221 inhibitor, or miR-222 inhibitor. (**B**) Quantitation of wound closure rates at different time points. (**C**) Transwell assay shows that the cells penetrated the insert membrane. (**D**) **Quantitation of the n**umber of cells that penetrated the insert membrane. Data represent the mean value ± SEM from three separate experiments. ^*,#^ P<0.05 vs pEX-5, ^**,##^ P<0.01 vs pEX-55).

As shown in Figures 5A and B, the closure rates of the miR-221 inhibitor and miR-222 inhibitor groups were lower than was that of pEX-5 group. For instance, the closure rates were 25±1.5% and 34±2.9% for miR-221 and miR-222 inhibitors, respectively, versus 57±7% for pEX-5 (P<0.01 to 0.05) at 48 h; and 47±2.7% and 59±9.9% versus 100% (P<0.01) at 72 h. Similar findings for inhibition of migration were observed using a transwell assay. As shown in Figures 5C and D, cell migration was significantly decreased after transfection with miR-221 inhibitor or miR-222 inhibitor compared with in cells transfected with pEX-5 (P<0.01).

### Inhibition of mir-221 and mir-222 increase sirt1 protein expression

**Figure 6:**
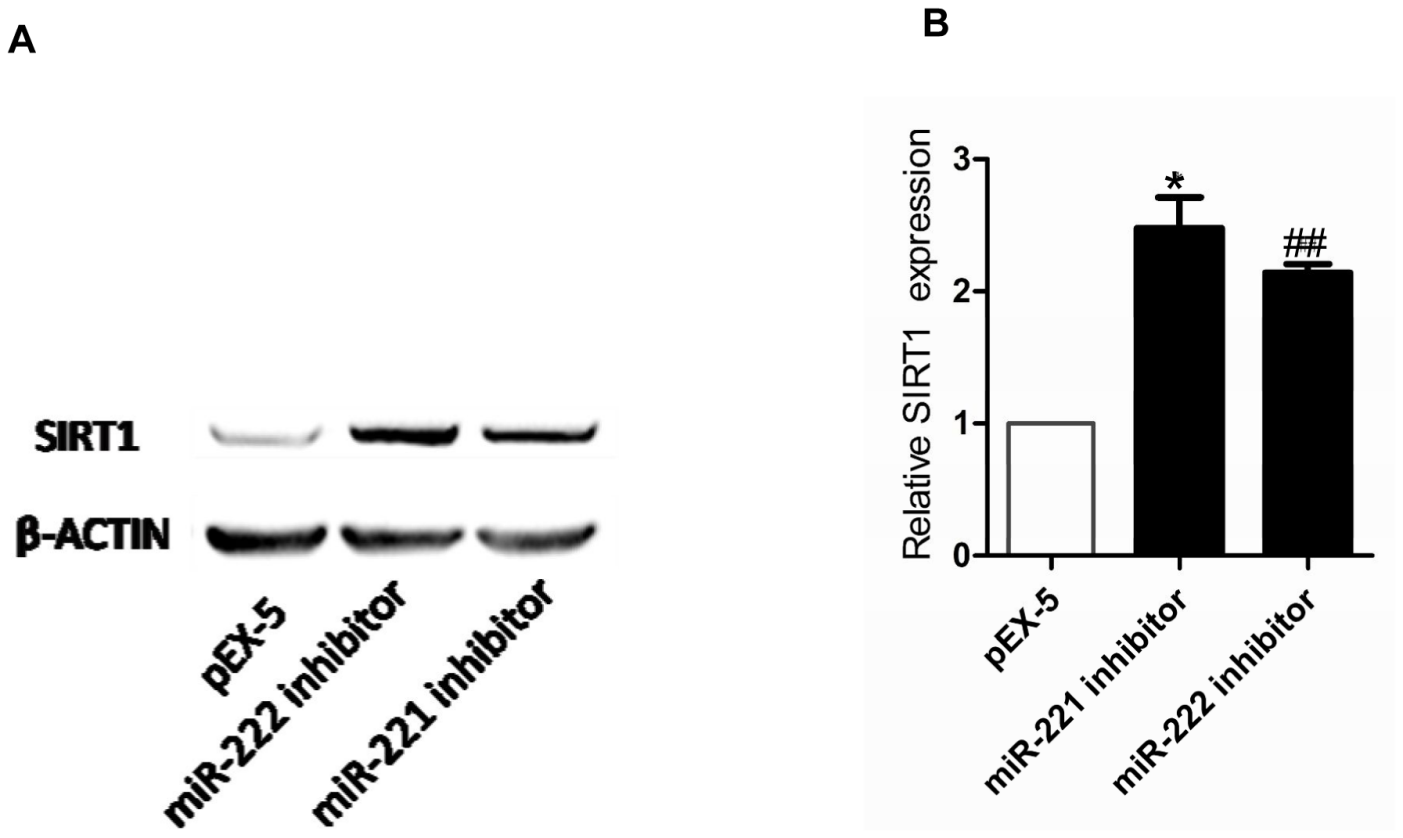
Effect of miR-221 inhibitor or miR-222 inhibitor on expression of SIRT1 protein. (**A**) Expression of SIRT1 protein levels in PC-3 cells after transfection were determined by Western blot analysis. (**B**) Quantitation of SIRT1 protein expression. Data represent the mean value ± SEM from three separate experiments. ^*^P<0.05 vs pEX-5, ^##^P<0.01 vs pEX-5.

Western blot analysis demonstrated that the SIRT1 protein levels of cells transfected with miR-221 inhibitor and miR-222 inhibitor were markedly increased compared with those of cells transfected with pEX-5 (P<0.05 and P<0.01, respectively) (Figures 6A and B). However, there were no significant differences in mRNA levels (Date was shown in Figure S2).

### Effects of sirt1 on proliferation, apoptosis, and migration in pc-3 cells

**Figure 7:**
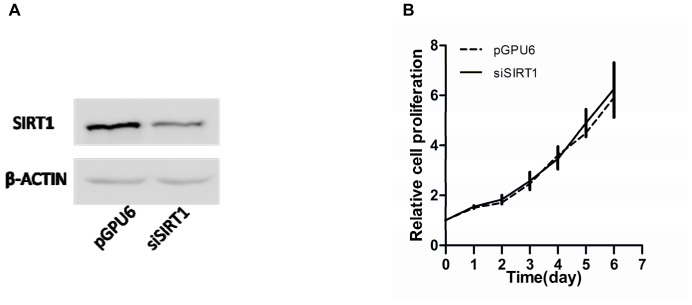
Effect of down-regulation of SIRT1 on PC-3 cell proliferation. (**A**) SIRT1 protein levels in PC-3 cells transfected with pGPU6 (empty vector plasmid) or siSIRT1 (pGPU6-si-SIRT1) by Western blot analysis. (**B**) Cell proliferation was quantitated using a CCK-8 assay. There were no significant differences between the two groups (P>0.05). Data represent the mean value ± SEM from three separate experiments.

**Figure 8:**
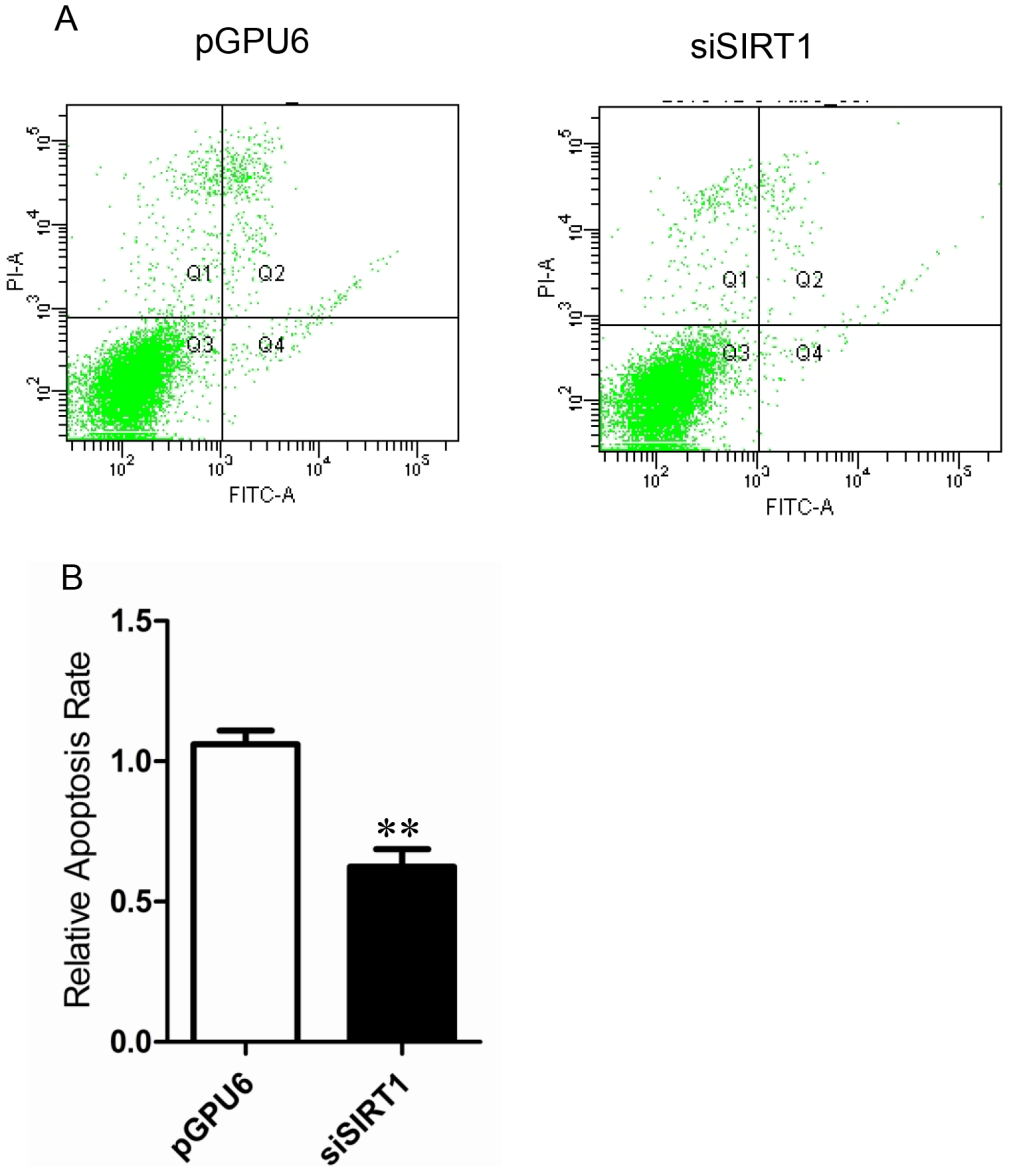
Effect of down-regulation of SIRT1 on apoptosis. (**A**) Apoptosis was measured by flow cytometry inPC-3 cells transfected with pGPU6 or pGPU6-siSIRT1. (**B**) Relative apoptosis rate of each group. Data represent the mean value ± SEM from three separate experiments. ^**^P<0.01 vs pGPU6.

**Figure 9:**
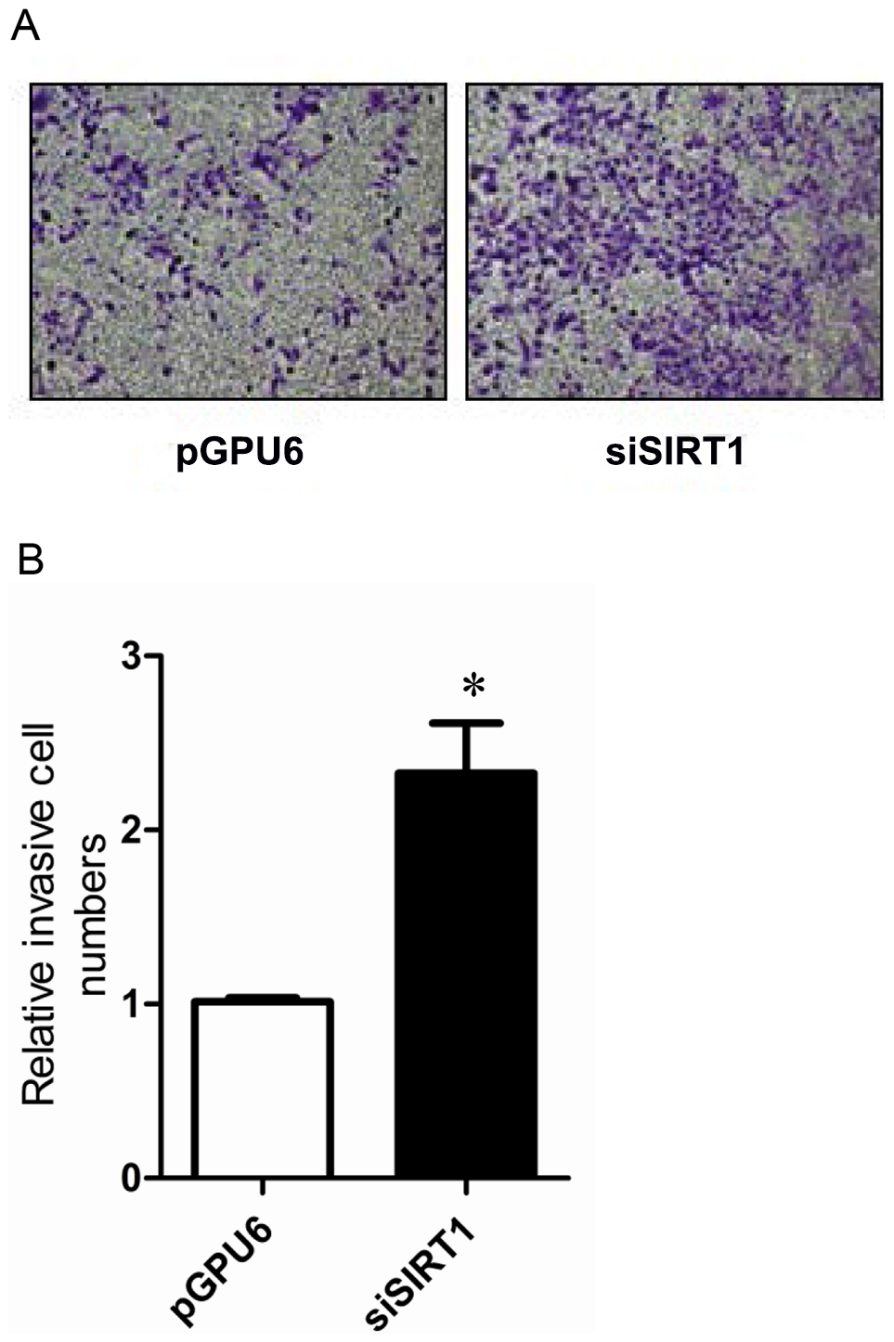
Effect of down-regulation of SIRT1 on PC-3 cell migration. (**A**) A transwell assay was used to measure the migration of cells transfected with pGPU6 or pGPU6-siSIRT1. (**B**) Number of cells penetrated the insert membrane. Data represent the mean value ± SEM from three separate experiments. ^*^P<0.05 vs pGPU6.

SIRT1 expression was significantly suppressed in cells transfected with siSIRT1 compared with those transfected with pGPU6 as a control (Figure 7A). There were no significant differences in cell proliferation between cells transfected with siSIRT1 and pGPU6 (Figure 7B). However, cells transfected with siSIRT1 resulted in a two-fold reduction in the apoptosis rate compared with that in cells transfected with pGPU6 (P<0.01) (Figures 8A and B). Interestingly, the migration of cells transfected with siSIRT1 was significantly increased compared with that of cells transfected with pGPU6 (P<0.05) (Figures 9A and B).

### Sirt1 is not a direct target of mir-221 and mir-222

**Figure 10:**
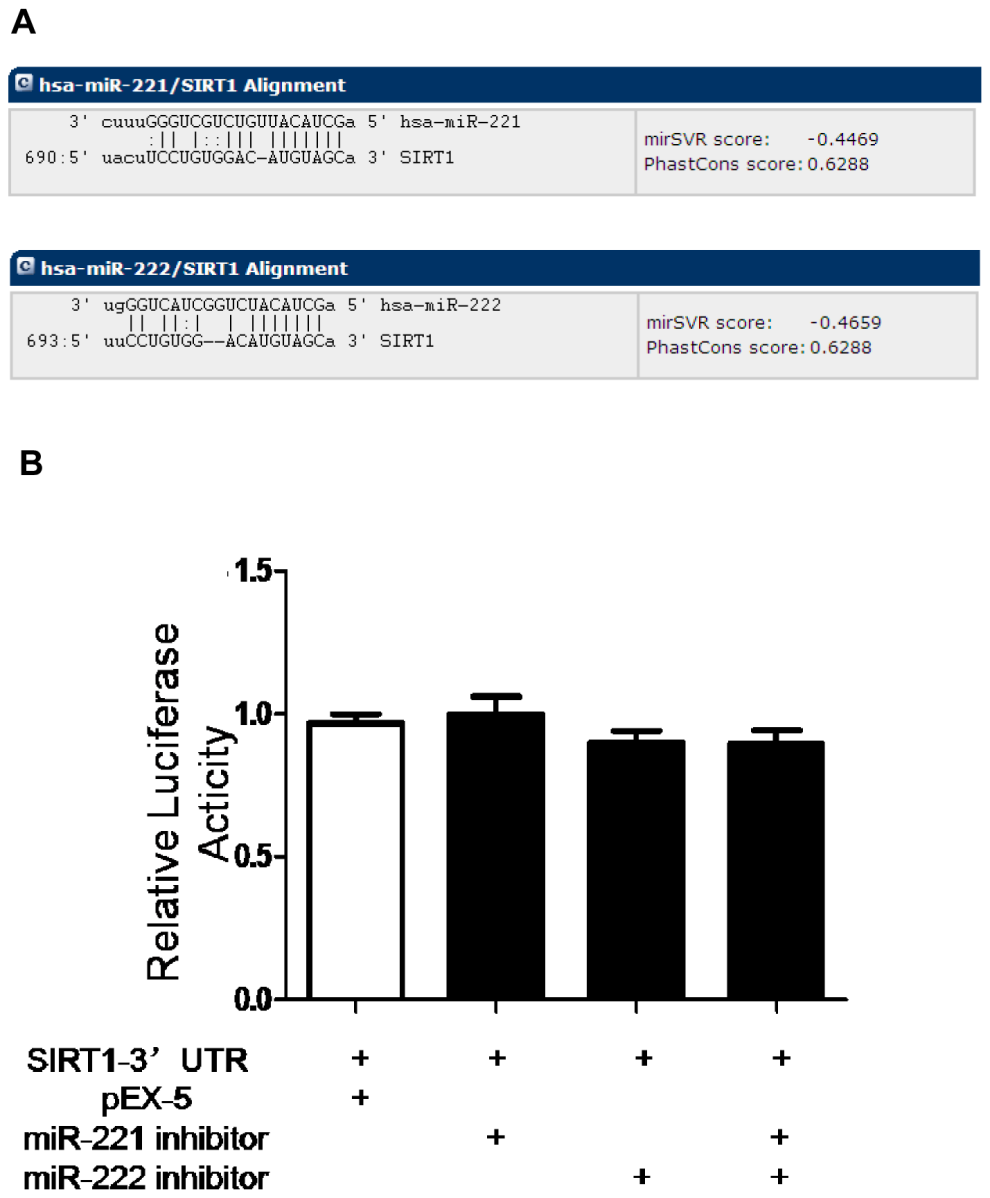
Regulation of SIRT1 by miR-221 and miR-222. (**A**) The complementary binding sites of miR-221 and miR-222 on SIRT1-3′UTR predicted by miRanda database. (**B**) Luciferase activity was carried out to verify the binding and interaction of miR-221 and miR-222 with SIRT1. There were no differences in luciferase activity among the groups (P>0.05). Data represent the mean value ± SEM from three separate experiments.

As the miRanda data show, miR-221 and miR-222 bound to one of the target sequences located in the 3′-UTR of SIRT1 mRNA (Figure 10A). To ascertain the direct interaction between miR-221 or miR-222 and SIRT1 mRNA-3′UTR, luciferase activity assay was conducted. However, luciferase activities revealed no statistically significant differences in miR-221 or miR-222 level in PC-3 cells co-transfected with SIRT1-3′UTR binding sequence reporter plasmid compared with the control (Figure 10B). In other words, miR-221 and miR-222 did not bind to the SIRT1 sequence directly and did not exert a direct biological interaction.

## Discussion

Several studies have shown that miR-221 and miR-222 are up-regulated in various cancers and are thus considered oncogenes [9],[13],[29]. Indeed, studies have found that miRNAs affect a series of biological processes through complementary binding to one or several target genes. For instance, p27, TRPS1, PTEN, ARHI, TIMP3, HECTD2 and RAB1A were the target genes of miR-221 and miR-222 [10],[11],. These findings suggest that these miRNAs are a therapeutic target. We have shown that the levels of miR-221 and miR-222 were significantly increased in PC-3 cells compared with in LNCap cells, which is consistent with other findings[34]. Down-regulation of miR-221 or miR-222 expression inhibited cell proliferation and migration and increased apoptosis in PC-3 cells. Moreover, effective transfection of miR-221 inhibitor or miR-222 inhibitor resulted in a higher percentage of cells in G0/G1 phases and a lower percentage of cells in S phase. These findings indicate that inhibition of miR-221 or miR-222 expression exerts important biological effects on cell proliferation, apoptosis, cell migration, cell cycle distribution, and cell transition in PC-3 cells. The expression of SIRT1 was increased in PC-3 cells after miR-221 and miR-222 were down-regulated. This finding suggests that SIRT1 plays a suppressive role against the tumor-promoting action of miR-221 and miR-222. SIRT1 has been reported to restrain LNCap cell proliferation [35]. Wang et al. demonstrated reduced SIRT1 expression in PCa tissue compared with in benign prostatic hyperplasia tissue [27]. SIRT1 can serve as a tumor promoter or tumor suppressor, depending on the oncogenic pathway specific to particular tumors [19],[22],[24]. SIRT1 might act as an oncogene by inhibiting tumor suppressor genes, such as p53 [36], and might act as a anti-oncogene by repressing several oncogenes or oncoproteins such as β-catenin and survivin [37],[38].

In our study, knocking down SIRT1 in PC-3 cells led to increased cell migration and a decreased apoptosis rate, but no significant difference was observed in cell proliferation. These findings suggest that SIRT1 participates in migration suppression and apoptosis promotion in PC-3 cells but has little role in regulating cell viability. Inhibiting miR-221 and miR-222 expression enhanced SIRT1 expression, suggesting that the observed increased apoptosis and decreased migration after transfection with miR-221 or miR-222 inhibitor is regulated by SIRT1 activation. These cells' proliferation-suppression capacity after miR-221 and miR-222 down-regulation may be modulated through other molecular pathways, such as p27[31] or ARHI[39]. Although SIRT1-3′UTR exists in the binding sites of miR-221 and miR-222, a luciferase reporter assay did not identify a direct binding relationship between SIRT1 and miR-221 or miR-222. This suggests that miR-221 and miR-222 indirectly restrain the expression of SIRT1 through other molecular pathways. Further studies are needed to illustrate the mechanisms and molecular pathways of miR-221, miR-222, and SIRT1 that are involved in the PCa pathogenesis by overexpressing the two microRNAs and modulating the expression of SIRT1 in different cell lines. Investigation of the biological functions of miR-221/222 and SIRT1 and their relationship in vivo using animal model should also be considered.

In summary, miR-221 and miR-222 are highly expressed in PC-3 cells. Inhibition of miR-221 or miR-222 expression reduces cell proliferation and migration and increases apoptosis in PCa cells. SIRT1 protein is up-regulated in cells after transfection of miR-221 or miR-222 inhibitor. Cells transfected with siSIRT1 show increased migration and a decreased apoptosis rate, but no effect on cell proliferation. These data indicate that the biological effects of miR-221 and miR-222 on prostate cancer cells are associated with SIRT1. Modulation of the expression of these molecules could affect tumorigenesis of prostate cancer. These findings may provide a potential therapeutic approach to prostate cancer.

## Supporting Information

Figure S1 **Authentication of cells transfected with miR-221 or miR-222 inhibitor by Western blot.** 1–8: numbered clones. Successful clones indicated by arrows were pooled together to verify the biological experiments. (TIF)

Figure S2 **Expression of SIRT1 mRNA.** Real time PCR was carried out to measure the SIRT1 mRNA changes in PC-3 cells transfected with pEX-5, miR-221 inhibitor or miR-222 inhibitor. There was no statistical difference among groups. (TIF)
