# Supplementary figures and images for: Down-Regulation of mir-221 and mir-222 Restrain Prostate Cancer Cell Proliferation and Migration That Is Partly Mediated by Activation of SIRT1
Source: PLoS One. 2014 Jun 3;9(6):e98833. doi: 10.1371/journal.pone.0098833 (PMC4043919; doi:10.1371/journal.pone.0098833)

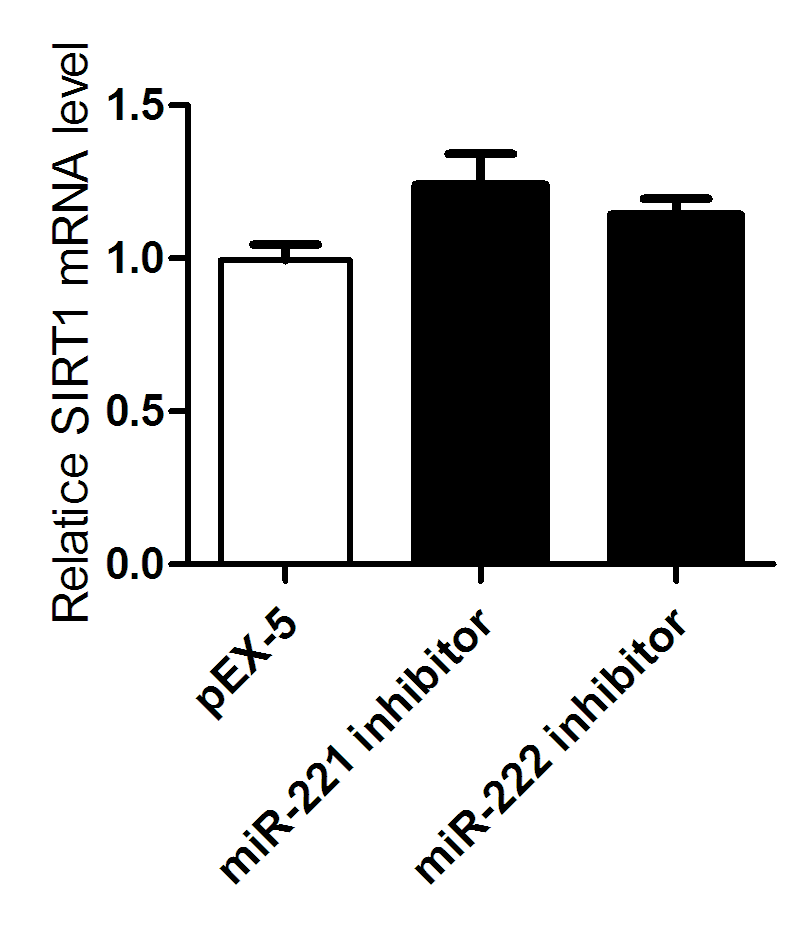

Supplement: Figure S1 — (TIF) [file pone.0098833.s001.tif]

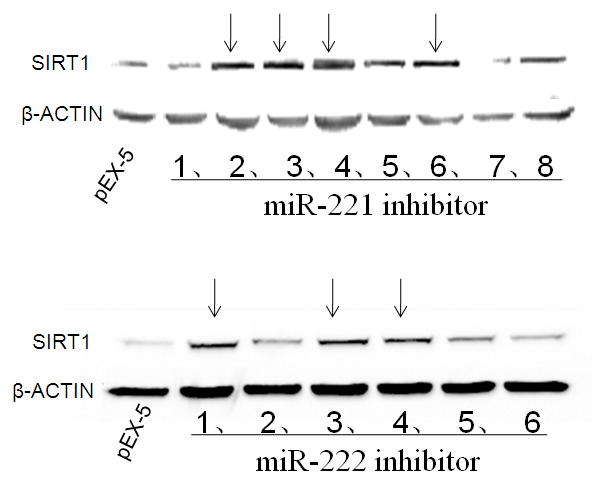

Supplement: Figure S2 — (TIF) [file pone.0098833.s002.tif]
